# Cooperation and Competition among information on social networks

**DOI:** 10.1038/s41598-020-69098-5

**Published:** 2020-07-22

**Authors:** Zhiqiang Zhu, Chang Gao, Yumeng Zhang, Hainan Li, Jin Xu, Yongli Zan, Zhi Li

**Affiliations:** 1 College of Science, Huazhong Agricultural University, Wuhan, 430070 China; 2 School of Mathematics, and School of Management, Shandong University, Jinan, 250353 China; 3 School of Mathematics and Statistics, Qilu University of Technology, Jinan, 250353 China

**Keywords:** Complex networks, Statistical physics

## Abstract

When multiple information are spread on social networks, there may be Cooperation and Competition among these information. Based on a new spreading model of multiple information, we studied Cooperation and Competition in information spreading, and analyzed the influence of different factors on Cooperation and Competition. Through a large number of computer simulation experiments, we found that: (1) when multiple information are spread on social networks, there is Cooperation and Competition among these information; (2) the smaller the distance between two information sources is, the stronger the Cooperation and Competition among these information are; (3) the greater the value of social reinforcement is, the stronger the Cooperation and Competition among these information are; (4) the weaker the human heterogeneity of one information is, the stronger the Cooperation and Competition among this information and other information are.

## Introduction

With the development of Internet technologies and the emergence of online social networks, the research of information spreading dynamics on complex networks has attracted a lot of attention from academia^1–11^. The emergence of online social networks^12^, on the one hand, is conducive to the information spreading, strengthening information communication and emotional communication between people^13^. For example, people use the social network to carry out viral marketing to promote their products, so that they are spreading faster and the people receiving information are more widely. On the other hand, the social network makes rumors, public opinions and other information spread quickly on the Internet. For example, the vast development of online social networks (*Twitter*, *Facebook*, *etc*.) has facilitated the spread of rumors in the population and made the influence of rumors much wider than ever before^14^.

Information are passed from one user to another through social networks. When people read newspapers, watch TV or browse news websites, they will constantly choose whether to accept certain media content. Once they received some information, they share posts with friends on social networks.

The widely adopted models of information spreading, such as Linear Threshold Model^15,16^, Generalized Threshold Model^17^, Independent Cascade Model^18^, all consider each information in isolation, independent of others. But the reality is that multiple information not only spread on the network at the same time, but also affect each other in the process of information spreading.

In the real world, there are usually multiple information spread on the network simultaneously. In addition, the information does not spread in isolation, depending on all other information currently spreading on the network. For example, we consider two news reports with similar content on the same event that are spread on the network at the same time. It is easy to see that these information would help each other in spreading, and many users would see these news reports. This would make them think the news report is real and very important, so they are more likely to accept and share it. In this case, these information help each other (“Cooperation”) in spreading. On the contrary, if we consider the two conflicting news reports on the same event, they would restrain each other in spreading. Because when users see these news reports, they have to choose to accept either or not. So in this case, these information inhibit each other (“Competition”) in spreading. Therefore, Competition reduces the possibility of spread, while Cooperation helps each other to be more widely spread on the network^19^.

In order to simulate information spreading more realistically, the model should include the interaction between information, so as to analyze the problems related to spreading dynamics^19–21^. We build a new model of information spreading in this paper, which can simulate multiple information spreading on the network at the same time, and there is interaction between information in the process of information spreading.

In recent years, a large number of scholars have done a lot of researches on the problem of spreading dynamics^22–24^, and some works related to this paper are as follows^25–32^.

Dodds and Watts^25^ introduced “memory effects” into the process of information spreading and found that “memory effects” affect the information spreading on the network. Centola^26^ used empirical research to find that the “social reinforcement” can affect the information spreading. Some researchers found that “social reinforcement” plays an important role in the spread of opinions, news, innovations and fads^27–30^. Lü et al.^31^ proposed a model to emphasize the difference between information spreading and epidemic spreading, and their model took into account of “memory effects”, “social reinforcement” and “non-redundancy of contacts”. Zhu et al.^32^ firstly put forward the concept of “human heterogeneity”, and proposed a model to emphasize the influence of human heterogeneity on information spreading, which includes four spreading mechanisms: (1) memory effects, (2) social reinforcement, (3) non-redundancy of contacts, (4) human heterogeneity.

In this paper, we study Cooperation and Competition in information spreading. Through computer simulation experiments on real social networks, we propose a spreading model of multiple information and analyze the interaction of information in information spreading. If two kinds of information are positive correlation, they will promote information spreading (that is, Cooperation) when they are spread on a network at the same time; but if they are negative correlation, they will inhibit information spreading (that is, Competition).

The models in Zhu et al.^32^ and other researches^15–18^ are only suitable for the spread of single information, or the spread of multiple information but the information spread in isolation. The model in this paper is based on the model in^32^ and adds the property of “interaction between information”. Our new model is suitable for the spread of multiple information on the network. By adjusting the value of parameter $\beta$ in the model, we can use computer to simulate that: when $\beta = 0$, we can simulate that multiple information spread independently on the network; when $\beta \ne 0$, we can simulate the spread of multiple information on the network, and there is interaction between information in the process of spreading. In addition, our spreading model considers four spreading mechanisms: (1) memory effects, (2) social reinforcement, (3) non-redundant contact, (4) human heterogeneity. Our model can be extended to the cases that three or more information spread on social networks at the same time.

The main contributions of our research to the field of spreading dynamics are as follows:

1. We propose a new spreading model. The model includes more comprehensive spreading mechanisms, which is not only applicable to the spread of multiple information on the network, but also can simulate the interaction of multiple information in the process of spreading. Therefore, this model is closer to the real process of spreading than other models^22–32^.
2. The main innovations of this study are as follows:

Based on the new information spreading model, the following important results can be obtained by adjusting the values of different parameters in the spreading model.

1. When multiple information are spread on social networks, there is Cooperation and Competition among these information.
2. Various factors (distance between two information sources, social reinforcement, human heterogeneity) have important influence on Cooperation and Competition in information spreading.

In order to simplify the expression, we use the term “simulation experiments” to express the experiments that simulate the information spreading on the network through the computer.

## Methods

Next, we mainly analyze the Cooperation and Competition among multiple information in the spreading by implementing two contents: (1) Building a new spreading model; (2) Based on the new spreading model, we conduct simulation experiments on real social networks to analyze the influence of various factors in information spreading on Cooperation and Competition.

In order to understand the model more deeply and systematically, the next two parts (“Basic Definitions” and “Process of Information Spreading”) explain some concepts and related contents in the model. Then, the background networks of the simulation experiments and how to analyze the influence of related factors on the Cooperation and Competition in information spreading are described in “Preparations for Simulation Experiments”.

**Table 1:** The definitions and characteristics of some related terminologies in the model.

Terminology	Symbol	Definition	Characteristics
Information attribute	$I_{L}$	It is a constant (value range from 0 to 1) that reflects the degree of difficulty in accepting information *L* by individuals	The smaller information attribute is, the easier individuals will accepted information *L*
Individual attribute	$I_{L}^{'}(v)$	It is a constant (value range from 0 to 1) that reflects the extent of acceptance for any individual *v* when *v* first hears *L*	The larger the individual attribute is, the easier information *L* will be accepted
The coverage ofinformation spreading	$\eta$	It is the proportion of individuals who accept information to all individuals (value range from 0 to 1)	
Correlation Coefficientbetween two kinds ofinformation	$\beta$	It is a constant (value range from 0 to 1): (1) $0<\beta \le 1(-1\le \beta <0)$, indicating that when an individual receives two information, one information can promote (restrain) the individual to accept another information; (2) $\beta =0$, indicating that two information do not affect each other	The greater the value of $\beta$ is, the easier the information is to spread
Memory effects	$M_{L}(v,t)$	The individual *v* has received the information *L* for $M_{L}(v,t)$ times until *t* time step. In the spreading process, as time goes on, once *v* receives *L*, the value of $M_{L}(v,t)$ is increased by 1	The greater the value of $M_{L}(v,t)$ is, the easier information *L* will be accepted
Social reinforcement	*c*	The constant $c\,(c > 0)$ reflects the influence of social reinforcement	The greater the value of *c* is, the easier the information is to spread
Non-redundant contact		In the spreading process, each edge on the network is used at most once	
Human heterogeneity		The difference of individual attribute reflects the intensity of human heterogeneity	The greater the fluctuations of individual attribute is, the stronger human heterogeneity is

### Basic definitions

Suppose that two kinds of information *A* and *B* are spreading on the network *G*. Each node represents an individual, and each edge represents the social relationship between two individuals. Table 1 shows the definitions and characteristics of some related terminologies in our model.

In order to analyze Cooperation and Competition in information spreading, $\beta$ is equal to 1, $-1$ and 0 in this paper. By comparing the coverage of information spreading when $\beta = 1$ and $\beta = 0$ ($\beta = -1$ and $\beta = 0$), we find that there is Cooperation (Competition) in information spreading when $\beta = 1$ and $\beta = 0$ ($\beta = -1$ and $\beta = 0$): The spread of one information on social network would promote (inhibit) the spread of another information.

### Process of information spreading

**Figure 1:**
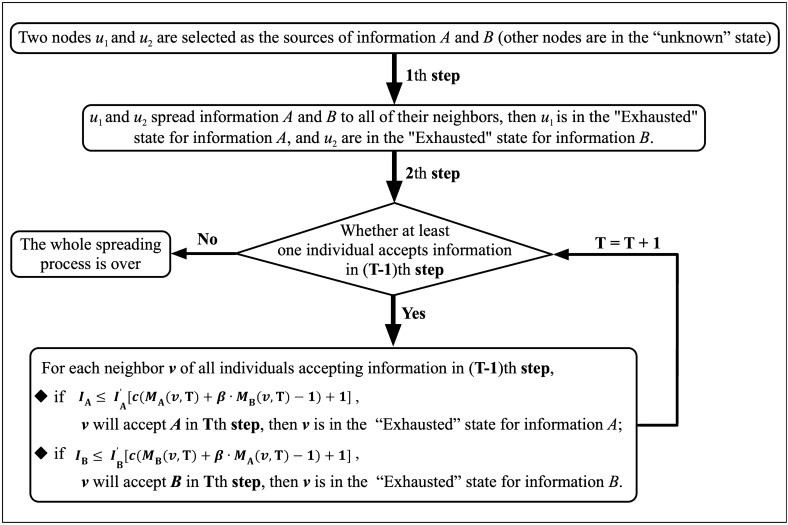
The spreading process of two kinds of information *A* and *B* on the network.

The spreading process of two kinds of information *A* and *B* on the network is shown in Fig. 1.

At the process of information spreading, each individual is in one of the four states:

1. “*Unknown*”: the individual has not yet come into contact with the information.
2. “*Known*”: the individual has come into contact with the information, but it does not spread to others because he is suspicious of the authenticity of information.
3. “*Accepted*”: the individual accepts the information and then spread it to all his neighbors.
4. “*Exhausted*”: after spreading the information to his neighbors, the individual will lose interest and never spread this information again.

The main process of information spreading are shown below:

At the beginning of information spreading, two nodes are randomly chosen as the spreading sources and the others are in the “Unknown” state.

**Figure 2:**
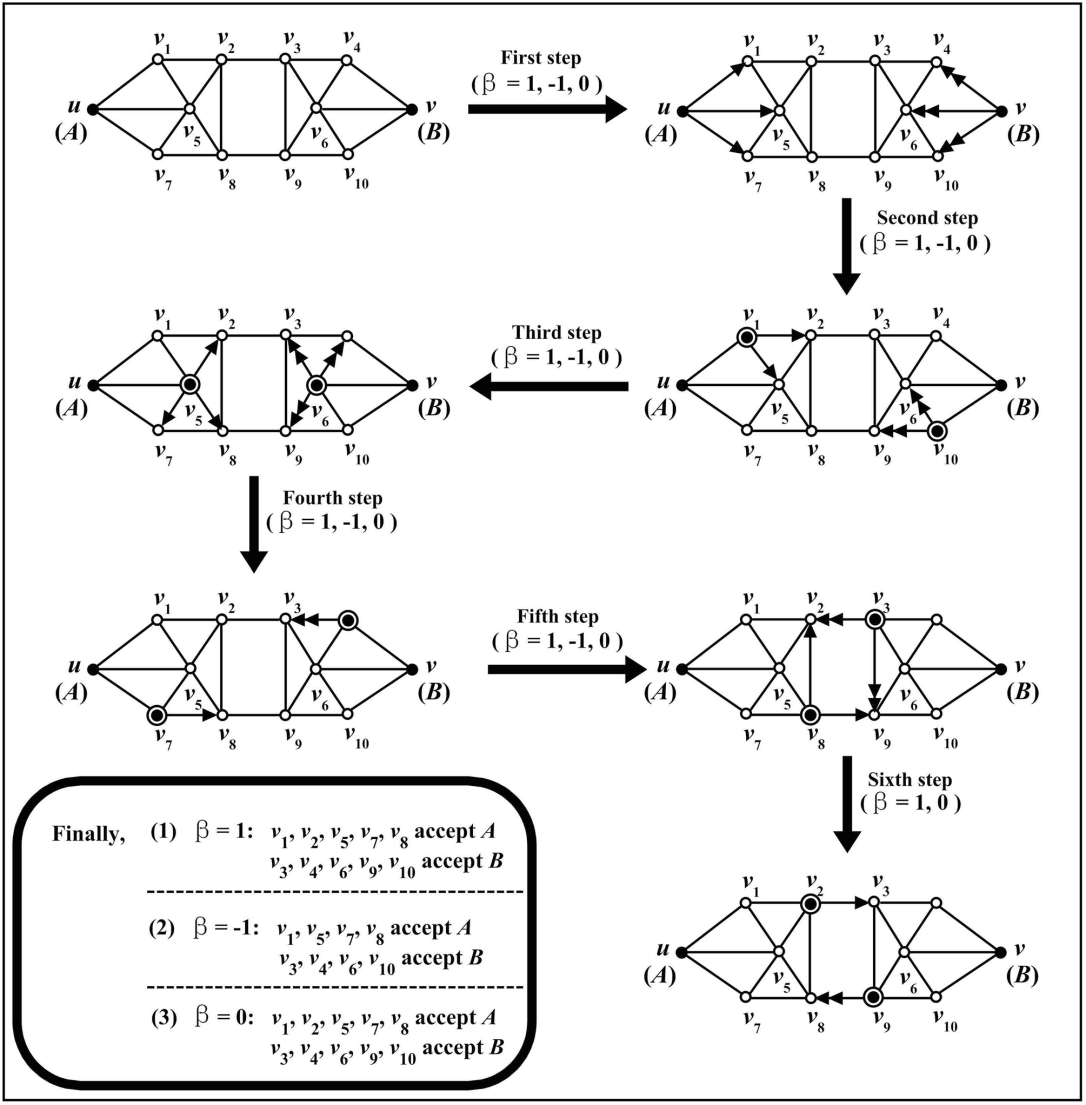
An example for the spreading process of information *A* and *B* on one network. Arrows indicate that information is spread from one node to another, and single arrow and double arrows represent the spread of *A* and *B*, respectively.

**Process 1**. (1th step) These two sources spread the information to all of their neighbors, then become “Exhausted” state.

**Process 2**. If there are new individuals accepting information in *T*th step ($$T \ge 1$$), the information will continue to spread, that is, these individuals will spread information to their neighbors at the same time in ($$T + 1)$$th step; otherwise, the whole spreading process is over.

Suppose that at least one individual accepts information in ($$T - 1)$$th step ($$T \ge 2$$), and individual *v* is one of their neighbors whose state is “Unknown” or “Known”, then the individual judges whether to accept the information according to the following methods:

- If $I_{A} \le I_{A}^{'}(v)[c(M_{A}(v, T) + \beta \cdot M_{B}(v, T) - 1) + 1]$, individual *v* will accept information *A* in *T*th step and spread information *A* to all his neighbors in (*T* + 1)th step, and *v* is in the “Exhausted” state for information *A*; otherwise, *v* do nothing no matter how many times this individual has received information *A*.
- If $I_{B} \le I_{B}^{'}(v)[c(M_{B}(v, T) + \beta \cdot M_{A}(v, T) - 1) + 1]$, the conclusion of whether individual *v* accepts information *B* is similar to that in (a).

However, if individual *v* does not receive information *A* and *B* in the *T*th step, he will do nothing in (*T* + 1)th step.

In order to understand the above process of information spreading, we give one example to illustrate this process (see Fig. 2). Figure 2 is an example for the spreading process of information *A* and *B*.

Firstly, *u* and *v* are the nodes where information *A* and *B* begin to spread, respectively.

Let $$I_{A}=I_{B}=0.6,$$ $$I_{A}^{'}(v_{1})=I_{B}^{'}(v_{1})=0.8,$$ $$I_{A}^{'}(v_{2})=I_{B}^{'}(v_{2})=0.2,$$ $$I_{A}^{'}(v_{3})=I_{B}^{'}(v_{3})=0.3,$$ $$I_{A}^{'}(v_{4})=I_{B}^{'}(v_{4})=0.5,$$ $$I_{A}^{'}(v_{5})=I_{B}^{'}(v_{5})=0.4,$$ $$I_{A}^{'}(v_{6})=I_{B}^{'}(v_{6})=0.4,$$ $$I_{A}^{'}(v_{7})=I_{B}^{'}(v_{7})=0.5,$$ $$I_{A}^{'}(v_{8})=I_{B}^{'}(v_{8})=0.3,$$ $$I_{A}^{'}(v_{9})=I_{B}^{'}(v_{9})=0.2,$$ $$I_{A}^{'}(v_{10})=I_{B}^{'}(v_{10})=0.8.$$

Then, the spreading process includes five steps (when $\beta = -1$) or six steps (when $\beta = 1$ or 0).

**First step**: no matter what the value of $\beta$ is ($\beta = -1$, 1 or 0),

$$I_{A}^{'}(v_{1})=0.8>0.6=I_{A}$$, $$I_{B}^{'}(v_{4})=0.5<0.6=I_{B}$$, $$I_{A}^{'}(v_{5})=0.4<0.6=I_{A}$$, $$I_{B}^{'}(v_{6})=0.4<0.6=I_{B}$$, $$I_{A}^{'}(v_{7})=0.5<0.6=I_{A}$$, $$I_{B}^{'}(v_{10})=0.8>0.6=I_{B}$$.

Then, $v_{1}$ accepts *A*, $v_{10}$ accepts *B*.

**Second step**: no matter what the value of $\beta$ is ($\beta = -1$, 1 or 0),

$$I_{A}^{'}(v_{2})=0.2<0.6=I_{A}$$, $$I_{A}^{'}(v_{5})\times [1\times (2-1)+1]=0.4\times 2=0.8>0.6=I_{A}$$, $$I_{B}^{'}(v_{9})=0.2<0.6=I_{B}$$, $$I_{B}^{'}(v_{6})\times [1\times (2-1)+1]=0.4\times 2=0.8>0.6=I_{B}$$.

Then, $v_{5}$ accepts *A*, $v_{6}$ accepts *B*.

Similar to the calculation process of the **First step** and **Second step**, we can analyze the spreading results of the next four steps when $\beta = 1$ or 0, and the next three steps when $\beta = -1$. In order to briefly describe the processes, here we only give the final spreading results:

1. $\beta = 1$ or 0: $v_{1}$, $v_{2}$, $v_{5}$, $v_{7}$, $v_{8}$ accepts *A*, $v_{3}$, $v_{4}$, $v_{6}$, $v_{9}$, $v_{10}$ accepts *B*;
2. $$\beta = -1$$: $$v_{1}$$, $$v_{5}$$, $$v_{7}$$, $$v_{8}$$ accepts *A*, $$v_{3}$$, $$v_{4}$$, $$v_{6}$$, $$v_{10}$$ accepts *B*;

### Preparations for simulation experiments

Next, through a large number of simulation experiments based on the above spreading model, we analyze the influence of various factors on the Cooperation and Competition in the information spreading.

**Table 2:** Some topological characteristics of three background networks.

Networks	Node number	Edge number	Diameter	Average clustering coefficient
$G_{1}$	63,731	817,035	15	0.148
$G_{2}$	39,199	87,415	17	0.082
$G_{3}$	81,306	1,768,149	7	0.565

Many real social network data^33,34^ can be used to analyze the topological characteristics of social networks and conduct research on spreading dynamics .

In this paper, we mainly carry out simulation experiments on the following three networks in Table 2.

Some topological characteristics of these three networks are listed in Table 2.

1. Network $G_{1}$: A real social network^35^, which contains friendship data of Facebook users, comprises 63,731 nodes and 817,035 edges: a node represents a user and an edge represents a friendship between two users. The data set of network can be downloaded from the following websites: http://konect.uni-koblenz.de/networks/facebook-wosn-links
2. Network $G_{2}$: A social network from Filmtipset.se^37^ is a Swedish movie rating website. Nodes in the network are users of the website and edges represent friendship. The data set of network can be downloaded from the following websites: http://konect.uni-koblenz.de/networks/filmtipset_friend
3. Network $G_{3}$: This social network^38^ contains Twitter data, which comprises 817,035 nodes and 1,768,149 edges. The data set of network can be downloaded from the following websites: http://snap.stanford.edu/data/ego-Twitter.html

Then we analyze the simulation experiments of information spreading on network $G_{1}$, $G_{2}$ and $G_{3}$. Because there are too many simulation results on each network, and the corresponding simulation process and results are similar, we put the simulation results on network $G_{2}$ and $G_{3}$ into the Supplementary information.

## Results

Next, we choose the network $G_{1}$^35^ as the background network of information spreading, and perform our spreading model on $G_{1}$ to simulate and analyze the influence of three factors (distance (*d*) between two information sources, social reinforcement (*c*) and human heterogeneity) on Cooperation and Competition.

In order to more clearly explain the influence of various factors on Cooperation and Competition, we give some symbols and explain them in Table 3.

**Table 3:** The definition of some symbols in the paper.

Symbol	Definition
${\eta ^{0}}$	The coverage of information spreading on the network when $\beta =0$
${\eta ^{-1}}$	The coverage of information spreading on the network when $\beta =-1$
${\eta ^{1}}$	The coverage of information spreading on the network when $\beta =1$
${\Delta \eta ^{-1}}\,(=\eta ^{-1}-\eta ^{0}$)	Difference value of the coverage of information spreading on the network when $\beta =-1$ and $\beta =0$
${\Delta \eta ^{1}}\,(=\eta ^{1}-\eta ^{0}$)	Difference value of the coverage of information spreading on the network when $\beta =\,1$ and $\beta =0$

Obviously, $\Delta \eta ^{-1} < 0$ indicates that there is Competition among information in information spreading, while $\Delta \eta ^{1} > 0$ indicates that there is Cooperation among information in information spreading.

To discuss the influence of human heterogeneity in information spreading, we consider three cases on the distribution of individual attribute as follows.

**Table 4:** Statistical characteristics of individual attribute in network $G_{1}$ subject to different distribution.

Individual Attribute	Mean ($$M $$)	Standard Deviation ($$SD $$)	Coefficient of Variation ($$CV = SD / M $$)
$N(0.5,0.15^{2})$	0.50	0.15	0.30
*U*(0, 1)	0.50	0.29	0.58
*Pow*(0.25)	0.44	0.30	0.68
*Pow*(0.5)	0.36	0.30	0.83
*Pow*(0.75)	0.27	0.28	1.04
*Pow*(1.0)	0.19	0.24	1.26

Let $P\{I^{'}_{L}(v) = x\}$ denotes the probability that the individual attribute $I^{'}_{L}(v)$ of randomly selected individual *v* is equal to *x*.

**Case 1**: $I^{'}_{L}(v)$ obeys normal distribution $N(0.5,0.15^{2})$, *i.e* $I^{'}_{L}(v) \sim N(0.5,0.15^{2})$;

**Case 2**: $I^{'}_{L}(v)$ obeys uniform distribution *U*(0, 1), *i.e* $I^{'}_{L}(v) \sim U(0,1)$;

**Case 3**: Let $n \ge 2$ be an integer and *X* = $\{\frac{i}{n} | i = 1, 2, ..., n\}$. For any $x\,\in \,(0,1]$, there is an integer *i* ($1 \le i \le n$) such that $\frac{i-1}{n}\,<\,x \le \frac{i}{n}$ and $I^{'}_{L}(v)$ obeys power-law distribution with power exponent $\lambda$ ($\lambda \ge 1$), *i.e* $P\{I^{'}_{L}(v) = x\,|\,\frac{i-1}{n}\,<\,x \le \frac{i}{n}\} = ai^{-\lambda }$, and $a = 1/(1^{-\lambda }+2^{-\lambda }+\cdots +n^{-\lambda })$. In order to simplify the expression, in the following contents of this paper, the power-law distribution is recorded as $Pow(\lambda )$, where $\lambda$ is the power index.

Although there is no data on human heterogeneity, and it is not clear what kind of distribution individual attributes will satisfy, this paper selects three common distributions in the real world^36^: Normal distribution, Uniform distribution and Power Law distribution as the distribution of individual attributes to study the influence of human heterogeneity in information spreading.

For individual attribute, the degree of dispersion reflects the intensity of human heterogeneity: the greater the degree of dispersion is, the stronger the human heterogeneity is.

**Figure 3:**
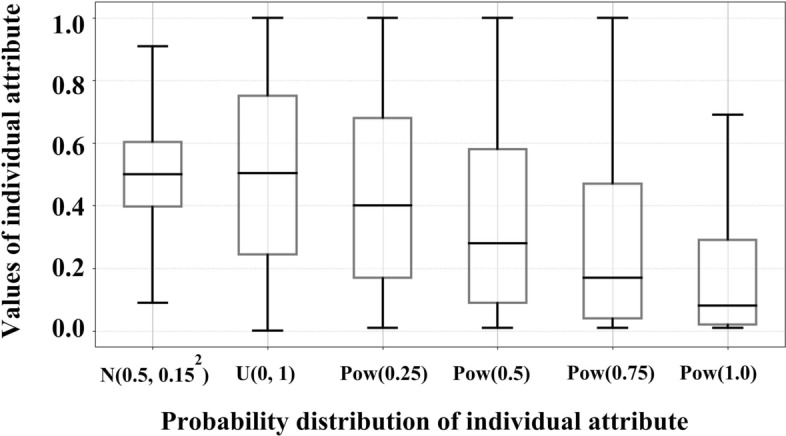
Box plot of individual attribute in network $G_{1}$ subject to different distribution.

There are two statistical variables used to measure the degree of dispersion:

1. Standard Deviation ($$SD $$) For a single set of data, the Standard Deviation can reflect its dispersion. The greater the Standard Deviation is, the greater the degree of dispersion is.
2. Coefficient of Variation ($$CV $$) For two or more sets of data, the Coefficient of Variation can be used to compare their dispersion. The greater the Coefficient of Variation is, the greater the degree of dispersion is. The Coefficient of Variation is equal to the ratio of Standard Deviation to Mean.

Therefore, for the sample data of individual attribute, we calculate the Coefficient of Variation to compare the intensity of human heterogeneity: the greater the Coefficient of Variation is, the stronger the human heterogeneity is; otherwise, the smaller the Coefficient of Variation is, the weaker the human heterogeneity is.

In order to analyze the influence of human heterogeneity, we consider that individual attribute $I^{'}_{L}(v)$ obey six probability distributions in this paper. Using the above conclusion “the greater the Coefficient of Variation is, the stronger the human heterogeneity is” and the data of Coefficient of Variation in Table 4, the order of human heterogeneity in these distributions is:

$$Pow(1.0)> Pow(0.75)> Pow(0.5)> Pow(0.25)> U(0,1) > N(0.5,0.15^{2})$$.

As shown in Fig. 3, it is the box plot of individual attribute in network $G_{1}$ subject to different distributions.

Next, the simulation experiments to analyze the influence of factors on Cooperation and Competition are divided into three parts “The influence of distance between two information sources”, “The influence of social reinforcement”, “The influence of human heterogeneity”.

### The influence of distance between two information sources

We firstly analyze the influence of distance between two information sources on the Cooperation and Competition in information spreading.

#### Design of simulation experiments in Fig. 4

**Figure 4:**
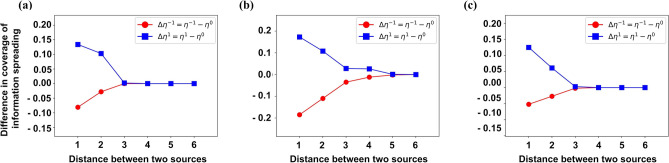
The influence of distance between two sources on Cooperation and Competition in information spreading. The parameters setting in the simulation experiments is: (**a**) $c = 1.0$, $I^{'}_{A}(v) \sim N(0.5,0.15^{2})$, $I^{'}_{B}(v) \sim N(0.5,0.15^{2})$; (**b**) $c = 1.0$, $I^{'}_{A}(v) \sim U(0,1)$, $I^{'}_{B}(v) \sim U(0,1)$; (**c**) $c = 1.0$, $I^{'}_{A}(v) \sim Pow(1.0)$, $I^{'}_{B}(v) \sim Pow(1.0)$. The greater the absolute value of $\Delta \eta ^{-1}$ ($\Delta \eta ^{1}$) is, the stronger the Cooperation (Competition) between information is.

When the individual attribute obeys certain distribution (such as Fig. 4a, $I^{'}_{A}(v) \sim N(0.5,0.15^{2})$ and $I^{'}_{B}(v) \sim N(0.5,0.15^{2})$), the following simulation experiments are carried out for each group of individual attribute generated by all individuals on the network:

1. Design of single simulation experiment: choose two nodes as two information sources, from these two nodes to spread information to their neighbors, and finally get the coverage of information spreading. For each pair of nodes, we carry out three such simulation experiment (in which the social reinforcement *c* is fixed, and $c = 1.0$ in Fig. 4): $\beta = 0$, $\beta = - 1$ and $\beta = 1$, and then calculate $\Delta \eta ^{-1}$ and $\Delta \eta ^{1}$.
2. Summary of results: all simulation results (i.e., the values of $\Delta \eta ^{-1}$ and $\Delta \eta ^{1}$) with the same distance (the distance between two sources) are accumulated and added respectively, and then the average value is taken.

Finally, 100 groups of individual attribute (i.e., the above process (1) and (2) are generated together for 100 times). Then all simulation results (i.e., the values of $\Delta \eta ^{-1}$ and $\Delta \eta ^{1}$) with the same distance (the distance between two sources) are cumulatively added and then the average value is taken.

Figure 4 presents the following results:

1. When the distance (*d*) between two information sources is small, $\Delta \eta ^{-1} < 0$ and $\Delta \eta ^{1} > 0$. So there is Cooperation and Competition in information spreading.
2. The smaller the distance (*d*) between two information sources is, the greater the absolute value of $\Delta \eta ^{-1}$ is, that is, the stronger the Competition between information is.
3. The smaller the distance (*d*) between two information sources is, the greater the absolute value of $\Delta \eta ^{1}$ is, that is, the stronger the Cooperation between information is.

### The influence of social reinforcement

**Figure 5:**
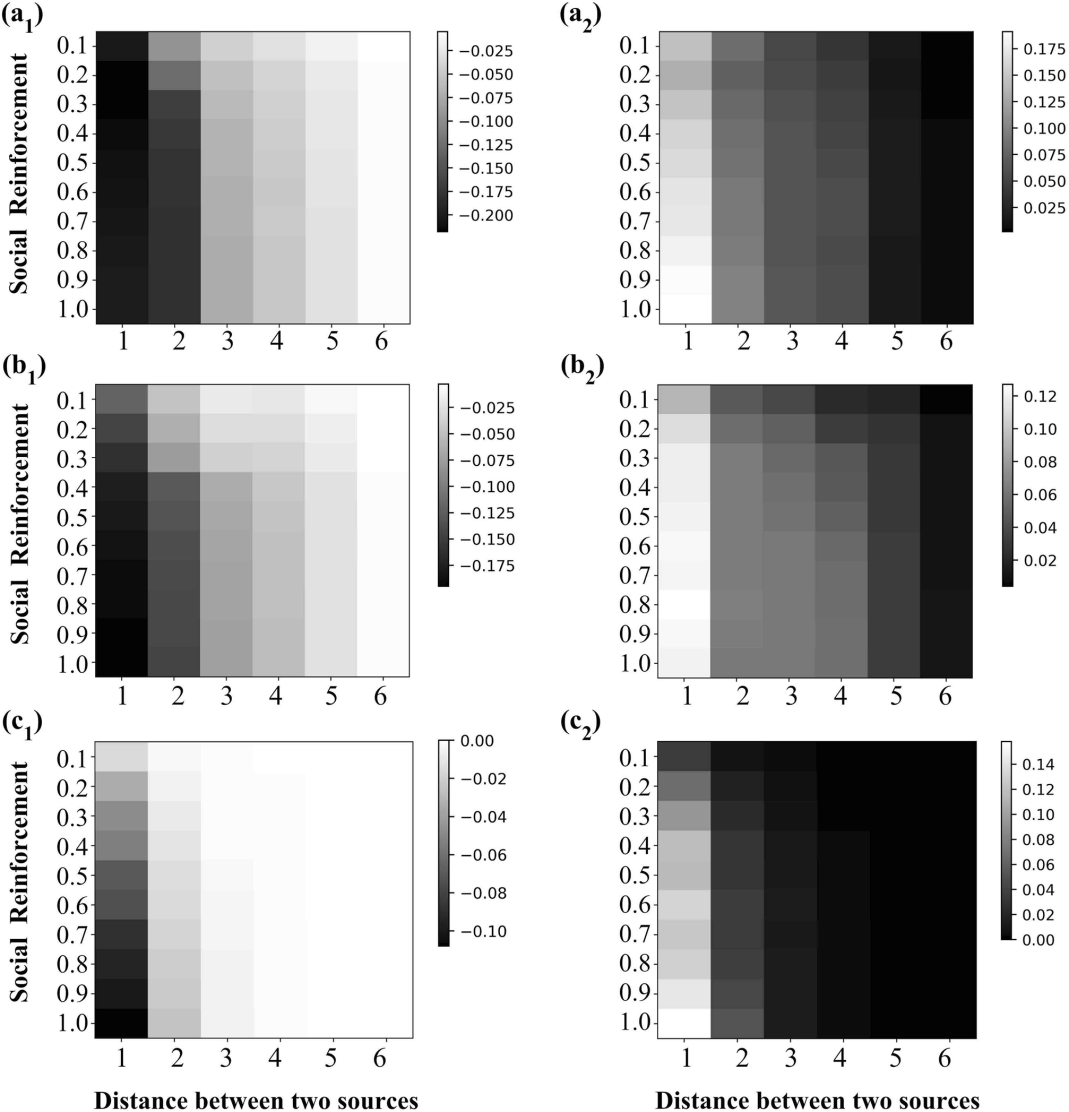
The influence of social reinforcement on Cooperation and Competition in information spreading. The parameters setting in the simulation experiment is following. (**a**$_{1}$) and (**a**$_{2}$): $I_{A}^{'} \sim N(0.5,0.15^{2})$, $I_{B}^{'} \sim N(0.5,0.15^{2})$; (**b**$_{1}$) and (**b**$_{2}$): $I_{A}^{'} \sim U(0,1)$, $I_{B}^{'} \sim U(0,1)$; (**c**$_{1}$) and (**c**$_{2}$): $I_{A}^{'} \sim Pow(1.0)$, $I_{B}^{'} \sim Pow(1.0)$. (**a**$_{1}$), (**b**$_{1}$) and (**c**$_{1}$): analyze the influence of social reinforcement on Competition (that is, to analyze the change of $\Delta \eta ^{-1}$), and the darker the color in the heat map (that is, the smaller the value of $\Delta \eta ^{-1}$ is), the stronger the Competition between information is; (**a**$_{2}$), (**b**$_{2}$) and (**c**$_{2}$): analyze the influence of social reinforcement on Cooperation (that is, to analyze the change of $\Delta \eta ^{1}$), and the lighter the color in the heat map (that is, the greater the value of $\Delta \eta ^{1}$ is), the stronger the Cooperation between information is.

Next, we analyze the influence of social reinforcement on the Cooperation and Competition in information spreading.

#### Design of simulation experiments in Fig. 5

When the individual attribute obeys certain distribution (such as Fig. 5 ($a_{1}$) and ($a_{2}$), $I^{'}_{A}(v) \sim N(0.5,0.15^{2})$ and $I^{'}_{B}(v) \sim N(0.5,0.15^{2})$), the following simulation experiments are carried out for each group of individual attribute generated by all individuals on the network:

1. Design of single simulation experiment: choose two nodes as two information sources, from these two nodes to spread information to their neighbors, and finally get the coverage of information spreading. For each value of social reinforcement *c* and each pair of nodes, we carry out three such simulation experiment: $\beta = 0$, $\beta = - 1$ and $\beta = 1$, and then calculate $\Delta \eta ^{-1}$ and $\Delta \eta ^{1}$.
2. Summary of results: all simulation results (i.e., the values of $\Delta \eta ^{-1}$ and $\Delta \eta ^{1}$) with the same distance (the distance between two sources) and the same social reinforcement are accumulated and added respectively, and then the average value is taken.

Finally, 100 groups of individual attribute (i.e., the above process (1) and (2) are generated together for 100 times).Then all simulation results (i.e., the values of $\Delta \eta ^{-1}$ and $\Delta \eta ^{1}$) with the same distance (the distance between two sources) and the same social reinforcement are cumulatively added and then the average value is taken.

Figure 5 presents the following results:

1. When the distance (*d*) between the two information is fixed, the greater the value of social reinforcement (*c*) is, the greater the absolute values of $\Delta \eta ^{-1}$ is; that is, the greater the value of *c* is, the stronger the Competition between information is.
2. When the distance (*d*) between the two information is fixed, the greater the value of social reinforcement (*c*) is, the greater the absolute values of $\Delta \eta ^{1}$ is; that is, the greater the value of *c* is, the stronger the Cooperation between information is.

### The influence of human heterogeneity

Finally, we analyze the influence of human heterogeneity on the Cooperation and Competition in information spreading. In order to analyze the influence of human heterogeneity in information spreading more systematically and deeply, we divide the simulation analysis into two parts: “The influence of human heterogeneity on spreading of single information” and “The influence of human heterogeneity on spreading of multiple information”.

#### The influence of human heterogeneity on spreading of single information

We first analyze the influence of human heterogeneity in information spreading when a single information is spread on the network.

In order to analyze the influence of human heterogeneity, individual attribute obeys six probability distributions in the following simulation experiments. From the above results, we find that the order of human heterogeneity in these distributions is: $Pow(1.0)> Pow(0.75)> Pow(0.5)> Pow(0.25)> U(0,1) > N(0.5,0.15^{2})$.

#### Design of simulation experiments in Fig. 6

We randomly select 50 nodes from the network, and carry out the following simulation experiments from each of these nodes:

1. When the individual attribute obeys a certain distribution, the simulation experiment is carried out for each group of individual attribute generated by all nodes of the network: a node as the information source, from this node to spread information to his neighbors, and finally get the coverage of information spreading in each step. 100 sets of individual attribute are generated together (i.e., the process is performed 100 times).
2. results summary: all simulation results (i.e., the values of $\eta$) with the same distribution for individual attribute and the same spreading time are cumulatively added and then the average value is taken.

**Figure 6:**
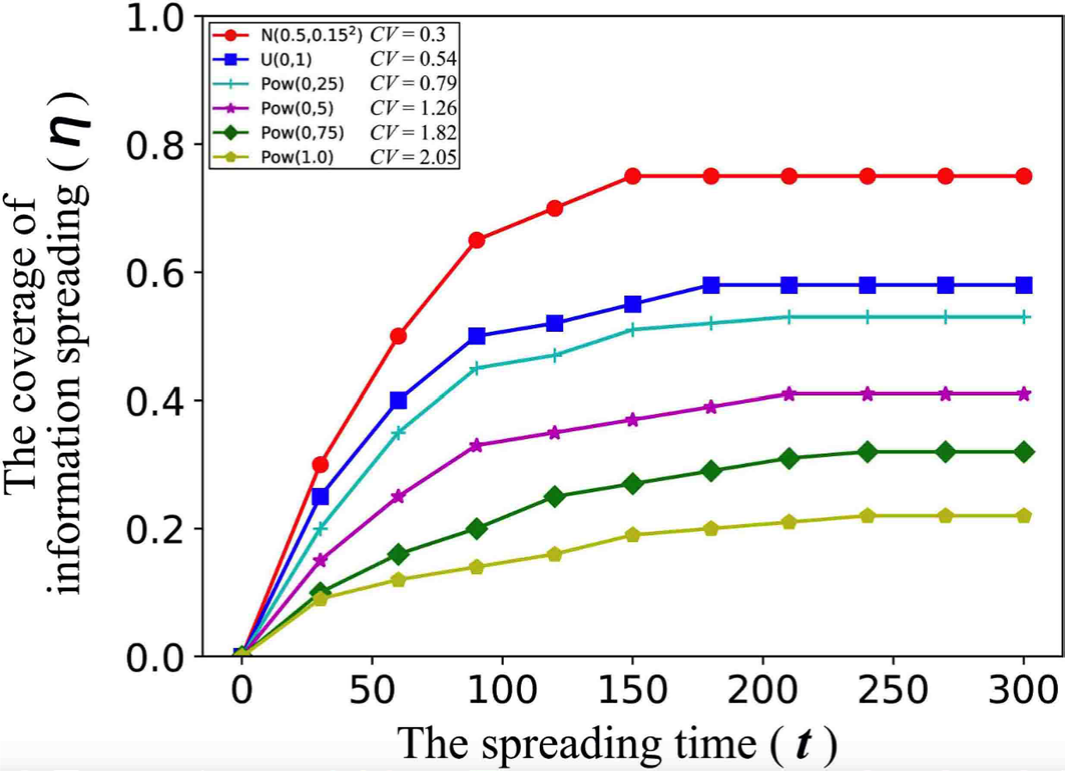
The influence of human heterogeneity in information spreading when a single information is spread on the network. The parameter setting in the simulation experiments is: $c = 1.0$.

According to Fig. 6, we find that: the stronger the human heterogeneity is (that is, the greater the Coefficient of Variation (*CV*) for individual attribute is), the smaller the coverage of information spreading is.

#### The influence of human heterogeneity on spreading of multiple information

Then we analyze the influence of human heterogeneity on the Cooperation and Competition in information spreading when two kinds of information are spread on the network.

#### Design of simulation experiments in Fig. 7

**Figure 7:**
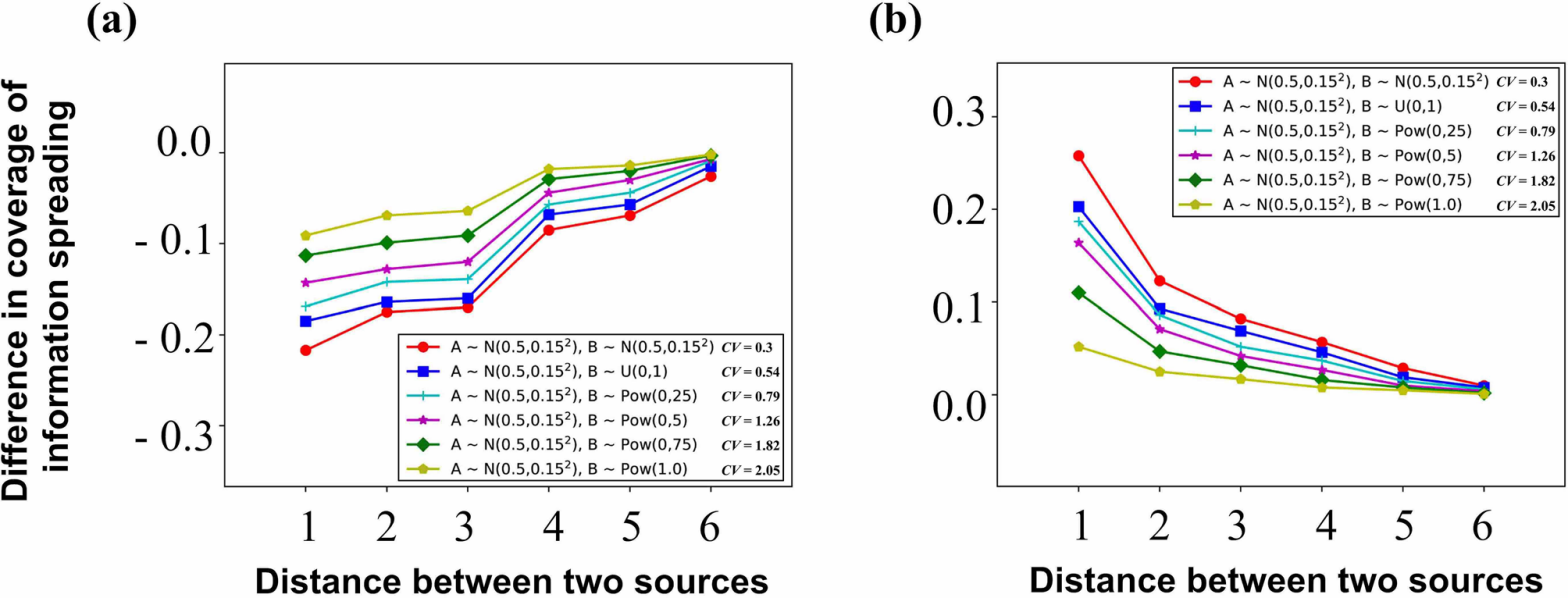
The influence of human heterogeneity on Cooperation and Competition in information spreading when two kinds of information are spread on the network. The parameter setting in the simulation experiment is: $c = 1.0$. (**a**) analyze the influence of social reinforcement on Competition (that is, to analyze the change of $\Delta \eta ^{-1}$); (**b**) analyze the influence of social reinforcement on Cooperation (that is, to analyze the change of $\Delta \eta ^{1}$).

When the individual attribute corresponding to one information is subject to a certain distribution (the distribution is fixed, such as $I^{'}_{A}(v) \sim N(0.5,0.15^{2})$ in Fig. 7), and the other information is subject to a certain distribution in six distributions, the following simulation experiments are carried out for each group of individual attribute generated by all individuals on the network:

1. Design of single simulation experiment: choose two nodes as two information sources, from these two nodes to spread information to their neighbors, and finally get the coverage of information spreading. For each pair of nodes, we carry out three such simulation experiment (in which the social reinforcement *c* is fixed, and $c = 1.0$ in Fig. 7): $\beta = 0$, $\beta = -1$ and $\beta = 1$, and then calculate $\Delta \eta ^{-1}$ and $\Delta \eta ^{1}$.
2. Summary of results: all simulation results (i.e., the values of $\Delta \eta ^{-1}$ and $\Delta \eta ^{1}$) with the same distance (the distance between two sources) are accumulated and added respectively, and then the average value is taken.

Finally, 100 groups of individual attribute (i.e., the above process is generated for 100 times).Then all simulation results (i.e., the values of $\Delta \eta ^{-1}$ and $\Delta \eta ^{1}$) with the same distance (the distance between two sources) are cumulatively added and then the average value is taken.

In order to further analyze the influence of human heterogeneity, in the following simulation experiments, the individual attribute of information *A* is subject to Normal distribution, while the individual attribute of information *B* is subject to Normal distribution, Uniform distribution, and Power Law distribution. So we can analyze the influence of *B* on *A* in information spreading, that is, the influence of different human heterogeneity on Cooperation and Competition. Figure 7 presents the following results:

1. When the social reinforcement (*c*) and distance (*d*) between two information are fixed, the greater the Coefficient of Variation (*CV*) of individual attribute for information *B*, the greater the absolute value of $\Delta \eta ^{-1}$ for information *A* is. That is, the weaker the human heterogeneity for one information is, the stronger the Competition between this information and other information is.
2. When the social reinforcement (*c*) and distance (*d*) between two information are fixed, the greater the Coefficient of Variation (*CV*) of individual attribute for information *B*, the greater the absolute value of $\Delta \eta ^{1}$ for information *A* is. That is, the weaker the human heterogeneity for one information is, the stronger the Cooperation between this information and other information is.

## Conclusions and discussion

In this paper, we study Cooperation and Competition in information spreading. Through simulation experiments on real social networks, we propose a new spreading model and analyze the influence of interaction between information in information spreading. We analyze the influence of three factors (distance between two information sources, social reinforcement, human heterogeneity) on Cooperation and Competition between two kinds of information.

Through a large number of simulation experiments, we find that:

1. When multiple information are spread on social networks, there is Cooperation and Competition between these information.
2. The smaller the distance (*d*) between two information sources is, the stronger the Cooperation and Competition between these information are.
3. The greater the value of social reinforcement (*c*) is, the stronger the Cooperation and Competition between these information are.
4. The weaker the human heterogeneity for one information is, the stronger the Cooperation and Competition between this information and other information are.

The results in this paper will help us to better understand the spread of multiple information on social networks, and the key factors that affect the Cooperation and Competition between multiple information, which can provide some theoretical guidance for the maximum information spreading (such as innovations, opinions, fads) and control of information spreading (such as rumors, viruses). In addition, although this paper only studies two kinds of information on social networks, our research methods and analysis ideas can be applied to the research of more than two kinds of information on social networks.

## Supplementary information

Supplementary information

## Data Availability

All network datasets in this study are available from KONECT (http://konect.uni-koblenz.de/networks/facebook-wosn-links, http://konect.uni-koblenz.de/networks/filmtipset_friend) and SNAP (http://snap.stanford.edu/data/ego-Twitter.html).
