# Supplementary material for: Cooperation and Competition among information on social networks
Source: Sci Rep. 2020 Jul 22;10:12160. doi: 10.1038/s41598-020-69098-5 (PMC7376155; doi:10.1038/s41598-020-69098-5)
Supplement: Supplementary file 1 — Supplementary information [file 41598_2020_69098_MOESM1_ESM.pdf]

The Interaction of Communication: Cooperation and Competition in Information Spreading

Zhiqiang Zhu[1,*], Chang Gao[1], Yumeng Zhang[1], Hainan Li[1], Jin Xu[2], Yongli Zan[3], Zhi Li[1]

[1]College of Science, Huazhong Agricultural University, Wuhan, 430070, China

[2]School of Mathematics, and School of Management, Shandong University, Jinan, 250353, China

[3]School of Mathematics and Statistics, Qilu University of Technology, Jinan, 250353, China

[*]Corresponding author

E-mail: zqzhu@mail.hzau.edu.cn

Supplementary Note 1: The simulation results of information spreading on network G_2

1.1 The background networks G_2

Network G_2: A social network from Filmtipset.se, a Swedish movie rating website. Nodes in the network are users of the website and links denote friendship. The data set of network can be downloaded from the following websites: http://konect.uni-koblenz.de/networks/filmtipset_friend

1.2 Simulation experiments on G_2

Supplementary Fig. S1: analysis of the influence of the distance (d) between the two information sources in information spreading;

Supplementary Fig. S2: analysis of the influence of social reinforcement (c) on Cooperation and Competition in information spreading;

Supplementary Fig. S3: analysis of the influence of human heterogeneity on information spreading when a single information is spread on the network G_2;

Supplementary Fig. S4: analysis of the influence of human heterogeneity on Cooperation and Competition in information spreading.

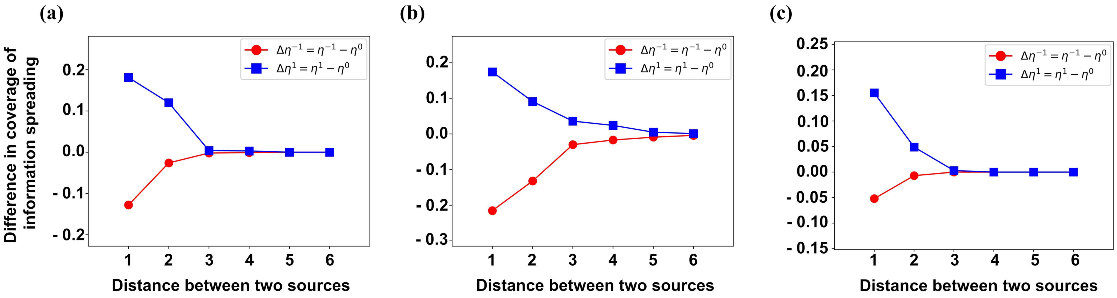

Supplementary Fig. S1. The influence of distance between two sources on Cooperation and Competition in information spreading. The parameters setting in the simulation experiments is: (a) $c = 1.0$, $I_A^{'}(v) \sim N(0.5, 0.15^2)$, $I_B^{'}(v) \sim N(0.5, 0.15^2)$; (b) $c = 1.0$, $I_A^{'}(v) \sim U(0, 1)$, $I_B^{'}(v) \sim U(0, 1)$; (c) $c = 1.0$, $I_A^{'}(v) \sim Pow(1.0)$, $I_B^{'}(v) \sim Pow(1.0)$. The greater the absolute value of $\Delta\eta^{-1}$ ($\Delta\eta^{1}$) is, the stronger the Cooperation (Competition) between information is.

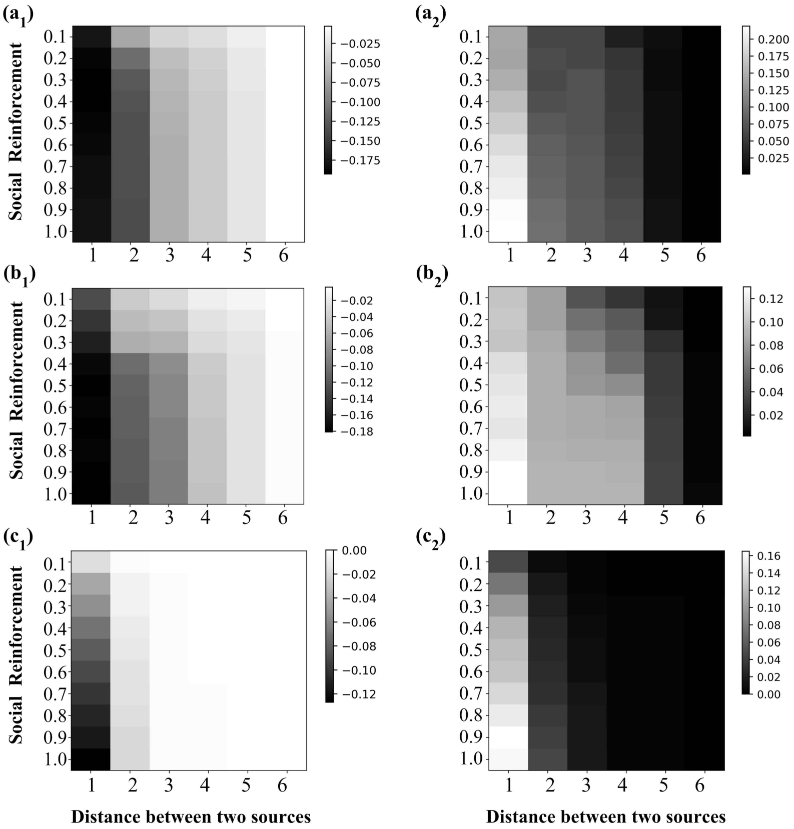

Supplementary Fig. S2. The influence of social reinforcement on Cooperation and Competition in information spreading. The parameters setting in the simulation experiment is following. (a_1) and (a_2): $I_A^{'} \sim N(0.5, 0.15^2)$,$I_B^{'} \sim N(0.5, 0.15^2)$; ($b_1$) and ($b_2$): $I_A^{'} \sim U(0, 1)$,$I_B^{'} \sim U(0, 1)$; (c_1) and (c_2): $I_A^{'} \sim Pow(1.0)$,$I_B^{'} \sim Pow(1.0)$. (a_1), (b_1) and (c_1) analyze the influence of social reinforcement on Competition (that is, to analyze the change of $\Delta\eta^{-1}$), and the darker the color in the heat map (that is, the smaller the value of $\Delta\eta^{-1}$ is), the stronger the Competition between information is; (a_2), (b_2) and (c_2) analyze the influence of social reinforcement on Cooperation (that is, to analyze the change of $\Delta\eta^{1}$), and the lighter the color in the heat map (that is, the greater the value of $\Delta\eta^{1}$ is), the stronger the Cooperation between information is.

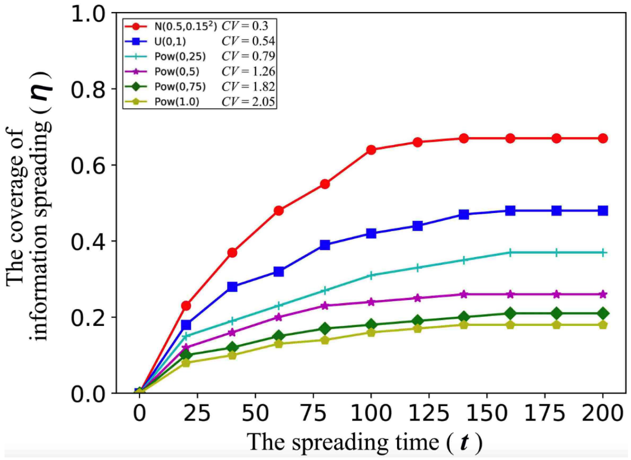

Supplementary Fig. S3. The influence of human heterogeneity on information spreading when a single information is spread on the network. The parameter setting in the simulation experiments is: $c = 1.0$.

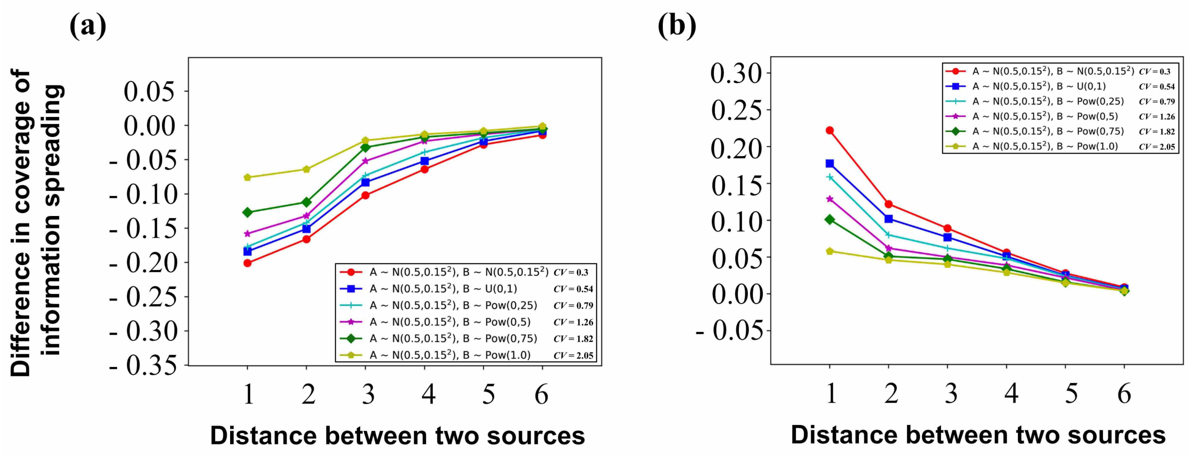

Supplementary Fig. S4. The influence of human heterogeneity on Cooperation and Competition in information spreading when two kinds of information are spread on the network. The parameter setting in the simulation experiment is: $c = 1.0$. (a): analyze the influence of social reinforcement on Competition (that is, to analyze the change of $\Delta\eta^{-1}$); (b): analyze the influence of social reinforcement on Cooperation (that is, to analyze the change of $\Delta\eta^{1}$).

Supplementary Note 2: The simulation results of information spreading on network G_3

2.1 The background networks G_3

Network G_3: This social network contains Twitter data, which comprises 817,035 nodes and 1,768,149 edges. The data set of network can be downloaded from the following websites: http://snap.stanford.edu/data/ego-Twitter.html

2.2 Simulation experiments on G_3

Supplementary Fig. S5: analysis of the influence of the distance (d) between the two information sources in information spreading;

Supplementary Fig. S6: analysis of the influence of social reinforcement (c) on Cooperation and Competition in information spreading;

Supplementary Fig. S7: analysis of the influence of human heterogeneity on information spreading when a single information is spread on the network G_3;

Supplementary Fig. S8: analysis of the influence of human heterogeneity on Cooperation and Competition in information spreading.

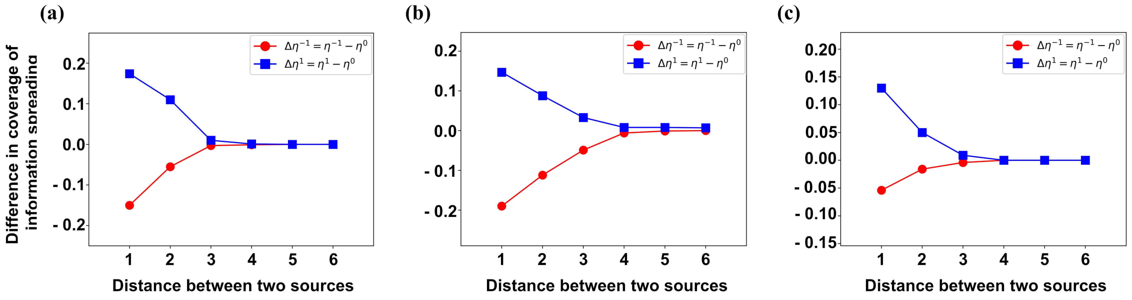

Supplementary Fig. S5. The influence of distance between two sources on Cooperation and Competition in information spreading. The parameters setting in the simulation experiments is: (a) $c = 1.0$, $I_A^{'}(v) \sim N(0.5, 0.15^2)$, $I_B^{'}(v) \sim N(0.5, 0.15^2)$; (b) $c = 1.0$, $I_A^{'}(v) \sim U(0,1)$, $I_B^{'}(v) \sim U(0,1)$; (c) $c = 1.0$, $I_A^{'}(v) \sim Pow(1.0)$, $I_B^{'}(v) \sim Pow(1.0)$. The greater the absolute value of $\Delta\eta^{-1}$ ($\Delta\eta^{1}$) is, the stronger the Cooperation (Competition) between information is.

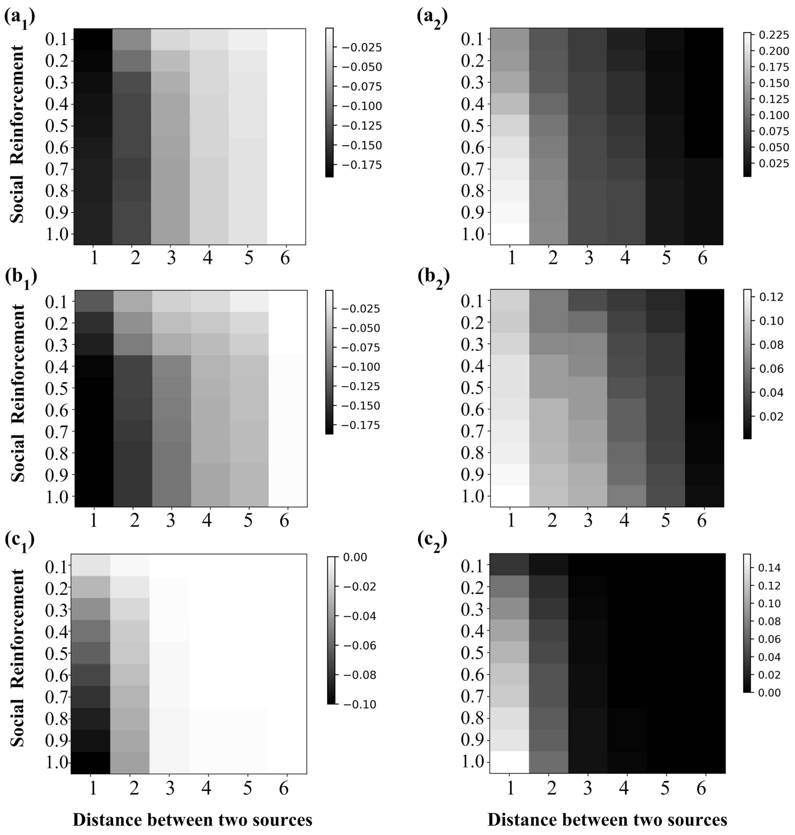

Supplementary Fig. S6. The influence of social reinforcement on Cooperation and Competition in information spreading. The parameters setting in the simulation experiment is following. (a_1) and (a_2): $I_A^{'} \sim N(0.5, 0.15^2)$,$I_B^{'} \sim N(0.5, 0.15^2)$; (b_1) and (b_2): $I_A^{'} \sim U(0,1)$,$I_B^{'} \sim U(0,1)$; (c_1) and (c_2): $I_A^{'} \sim Pow(1.0)$,$I_B^{'} \sim Pow(1.0)$. (a_1), (b_1) and (c_1) analyze the influence of social reinforcement on Competition (that is, to analyze the change of $\Delta\eta^{-1}$), and the darker the color in the heat map (that is, the smaller the value of $\Delta\eta^{-1}$ is), the stronger the Competition between information is; (a_2), (b_2) and (c_2) analyze the influence of social reinforcement on Cooperation (that is, to analyze the change of $\Delta\eta^{1}$), and the lighter the color in the heat map (that is, the greater the value of $\Delta\eta^{1}$ is), the stronger the Cooperation between information is.

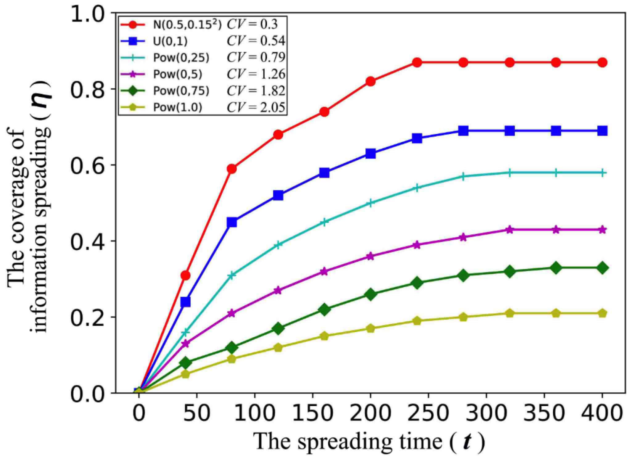

Supplementary Fig. S7. The influence of human heterogeneity on information spreading when a single information is spread on the network. The parameter setting in the simulation experiments is: $c = 1.0$.

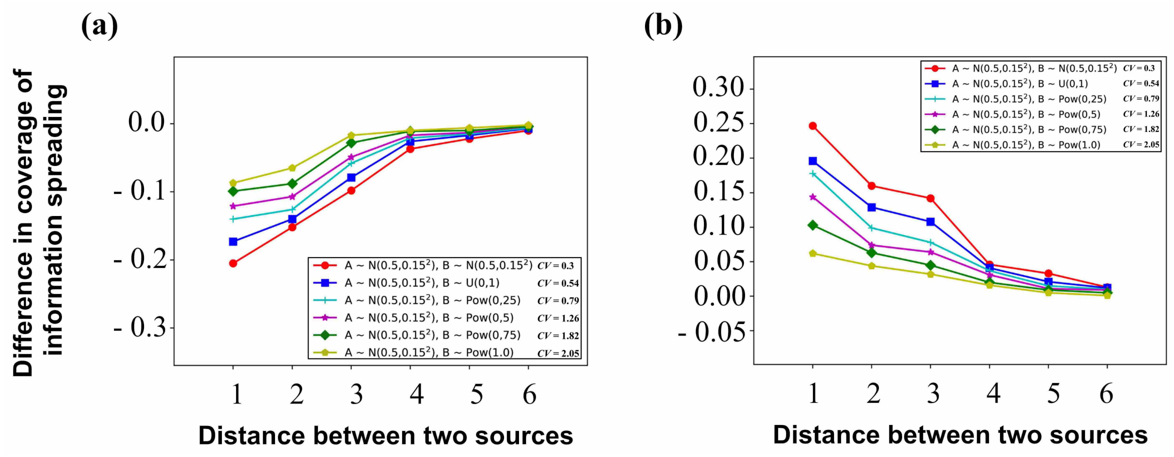

Supplementary Fig. S8. The influence of human heterogeneity on Cooperation and Competition in information spreading when two kinds of information are spread on the network. The parameter setting in the simulation experiment is: $c = 1.0$. (a): analyze the influence of social reinforcement on Competition (that is, to analyze the change of $\Delta\eta^{-1}$); (b): analyze the influence of social reinforcement on Cooperation (that is, to analyze the change of $\Delta\eta^{1}$).
